# Authors' Reply to: COVID-19 as a “Force Majeure” for Non–COVID-19 Clinical and Translational Research. Comment on “Analysis of Scientific Publications During the Early Phase of the COVID-19 Pandemic: Topic Modeling Study”

**DOI:** 10.2196/29156

**Published:** 2021-05-20

**Authors:** Andreas Älgå, Oskar Eriksson, Martin Nordberg

**Affiliations:** 1 Department of Clinical Science and Education, Södersjukhuset Karolinska Institutet Stockholm Sweden; 2 Department of Global Public Health Karolinska Institutet Stockholm Sweden; 3 DataRobot Inc Stockholm Sweden

**Keywords:** COVID-19, SARS-CoV-2, coronavirus, pandemic, topic modeling, research, literature, medical research, publishing, force majeure

We thank Petar Milovanovic and Igor Dumic [1] for raising the complex issue of challenges associated with non–COVID-19–related research. We share their view that many of the effects that the COVID-19 pandemic has had on such research are likely yet to be seen and that researchers and funders need to find pragmatic ways to deal with this new reality. However, some of the authors’ suggested actions, such as to adjust endpoints and power calculations, risk compromising research quality and should be done with caution. In times and settings associated with poor preconditions for clinical studies, we encourage researchers to assess the utility of alternative data sources, such as readily available medical image and routine clinical data.

We concur with the authors that analysis of open data and computer-based simulations have the potential to play a vital role in the future of medical research, apart from experimental and clinical studies. Additionally, we believe that methods like the one used in our study [2] may also prove to be a way forward. The number of published scientific studies is rapidly increasing, and traditional ways of compiling study results to generate new knowledge are slow and not suitable for these numbers. Therefore, the application of existing machine learning text analysis methods and the development of new ones holds promise of scientific gains not only from *existing data* but also from *already completed studies*.
